# State and federal policies and school meal participation: A descriptive analysis from Arizona

**DOI:** 10.1371/journal.pone.0350416

**Published:** 2026-06-12

**Authors:** Shreya Raval, Sarah Martinelli, Tatum Dykstra, Francesco Acciai, Juliana Cohen, Francesco Ramponi, Punam Ohri-Vachaspati

**Affiliations:** 1 College of Health Solutions, Arizona State University, Phoenix, Arizona, United States of America; 2 Department of Nutrition and Public Health, Merrimack College, North Andover, Massachusetts, United States of America; 3 Department of Nutrition, Harvard T.H. Chan School of Public Health, Boston, Massachusetts, United States of America; 4 Department of Global Health and Population, Harvard T.H. Chan School of Public Health, Boston, Massachusetts, United States of America; Jahangirnagar University, BANGLADESH

## Abstract

Starting in 2023, the state of Arizona implemented a series of policies to improve access to school meals: (1) A state policy to eliminate co-pays for reduced-price meals, implemented in January, 2023; (2) the federal Medicaid Direct Certification demonstration project, implemented in August, 2023; and (3) a federal policy to expand eligibility for Community Eligibility Provision (CEP), implemented in October, 2023. We track changes in school meal participation rates and number of meals served for breakfast and lunch after implementation of these policies using longitudinal data from 1,730 public and charter schools in Arizona. Outcomes were compared across different policy periods with baseline- the period prior to implementation of the three policies. Following the co-pay policy elimination, compared to baseline, participation among students eligible for reduced-price category increased significantly by about 10%, for both breakfast and lunch. After the implementation of all three policies, compared to baseline, student participation across all eligibility categories increased by 4.8% and 7.2% for breakfast and lunch, respectively. Similarly, compared to baseline, with all three policies in place, the average number of free meals served daily increased by 27.9% and 26.8% for breakfast and lunch, respectively. School CEP participation expanded from 451 to 737 schools- a 63% increase following the implementation of the lower eligibility threshold for CEP participation. As a result of increased school CEP participation, an additional 147,125 children had access to free school meals. Participation trends across eligibility categories varied by school urbanicity, grade level, and demographic characteristics of enrolled students. These findings suggest that implementing state and federal policies can enhance access to and participation in school meal programs.

## Introduction

School meals play an important role in school-age children’s diets [1]. Currently, 29.7 million children participate in the school lunch program and 15.5 million children in the school breakfast program each day on average [2]. Since the implementation of the Healthy Hunger-Free Kids Act of 2010 (HHFKA), school meals have become healthier and provide a greater amount and variety of fruits, vegetables, and more whole grains options than they did before [3–5]. In fact, school meals have been recognized as the healthiest source of food available to students outside of the home [6]. Further, the benefits of school meals can also be seen in the classroom, with improvements in student behavior and test scores [7–11].

School meals are funded primarily through the United States Department of Agriculture’s (USDA) “3-tiered system” [12], whereby, based on family income, students qualify for free, reduced, or paid meals. Students from families with income below 130% of the federal poverty line are eligible for free meals, those from families earning between 130–185% of the federal poverty line are eligible for reduced-price meals, while others are required to pay full price, at subsidized rates [12]. Additionally, the Community Eligibility Provision (CEP), introduced in 2014, allows schools serving a high proportion of low-income students to offer meals for free to all students, without requiring families to submit applications [13]. CEP requires schools to use the Identified Student Percentage (ISP)–the proportion of students directly certified to participate in free school meals based on their family’s participation in other means-tested programs, such as the Temporary Assistance to Needy Families or the Supplemental Nutrition Assistance Program–to determine eligibility [13]. CEP adoption saves school staff and parents time and is linked to increased school meal participation, improved academic outcomes, lower obesity prevalence, and reduced school meal debt [14–19].

In response to the COVID pandemic, in March of 2020, the USDA temporarily implemented Universal Free Meals (UFM), which allowed schools to serve free meals to all students regardless of family income. About one year and half later, in the fall 2022, UFM ended and most states reverted to CEP and the tiered funding model [20]. However, since late 2022, some states adopted policies to expand access to free meals [21]; for instance, Arizona eliminated co-pays for students eligible for reduced-price meals ($0.30 for breakfast and $0.40 for lunch) [22,23]. In addition to this state-level elimination of the reduced-price meal co-pay, two new federal policies were introduced that impacted access to school meals in Arizona. First, starting in the fall of 2023, Arizona capitalized on the USDA Medicaid Direct Certification demonstration program, allowing students from Medicaid-participating families to be deemed categorically eligible for free meals [24]. Second, in October 2023 USDA lowered the CEP eligibility threshold from an ISP of 40% or higher to an ISP of 25% or higher; Arizona applied for mid-year adoption, allowing more schools to participate in CEP starting January 2024 [25].

Previous studies have primarily focused on evaluating the impact of single policies, such as CEP or UFM [26,27]. A national study [28] examining the impact of CEP on NSLP participation showed a 6.8% increase in SY 2016−17 compared to non-participating schools, while another recent national study [29] found that states offering UFM showed consistently higher overall participation rates among enrolled students versus those without UFM. Our study builds on this prior work by examining sequential implementation of three policies. We track changes in school meal participation rates and number of meals served for breakfast and lunch after implementation of these federal and state policies implemented in Arizona between January 2023 and May 2024. Further, we assess differential effects of these policies on participation among students eligible for free, reduced-price, or paid meals as well as by school level factors. The study provides new evidence on how gradual, incremental (i.e., additional) policy strategies and their combination can expand access to school meals. These findings can help policymakers identify effective strategies to increase student participation in school meal programs in the absence of state level funding for UFM while also promoting equitable access to school meals.

## Materials and methods

### Study design

**Fig 1:**
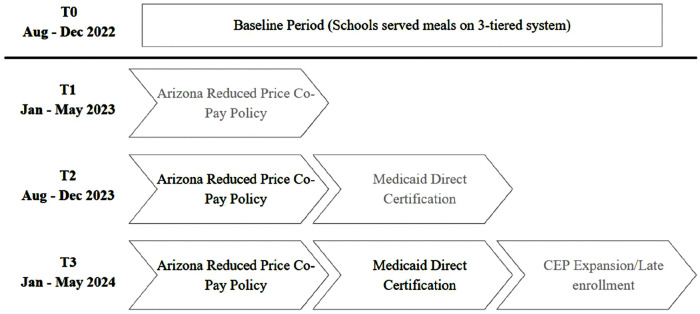
Study time period (T0-T3). *Shaded text represents the new policy implementation during that period. *Note: T0 or baseline (August-December 2022): Post-COVID and post-UFM, payment system returned to the 3-tiered system and CEP. T1 (January-May 2023): Arizona’s reduced-price co-pay policy was implemented, allowing students eligible for reduced-price meals to receive free meals. T2 (August-December 2023): Medicaid Direct Certification was introduced, enabling students from families receiving Medicaid to automatically qualify for free meals, with the Arizona reduced-price co-pay elimination policy already in place. T3 (January-May 2024): CEP eligibility threshold was lowered from 40% to 25% ISP, alongside Arizona’s reduced-price co-pay elimination policy and Medicaid Direct Certification.

A longitudinal study design was used to examine the impact of state and federal policy implementation on school meal participation in Arizona. The study was divided into four time periods (T0-T3) based on policy implementation, see Fig 1.

### Data sources

Data on CEP participation, meals served (breakfast and lunch) across eligibility categories (free, reduced-price, and paid), students’ eligibility, and school operational days were collected from the Arizona Department of Education (ADE) using Freedom of Information Act requests. Data were collected for a period starting in August 2022, when the federal UFM program ended, through May 2024. School-level variables such as locale, majority school race, and school level were drawn from the National Center for Education Statistics (NCES). The ADE data were accessed on 26th January, 2024, and the NCES data were obtained on 7th August, 2024.

Non-traditional schools, including boarding schools (n = 10), residential child care institutions (n = 26), Bureau of Indian Affairs schools (n = 33), juvenile detention centers (n = 11), and private non-residential schools (n = 41) were excluded from the sample, resulting in a final analytical dataset that included all public and charter schools in Arizona that operated the school meal programs (n = 1,730) in school years 2022−23 and 2023−24. Further, the months of June and July were excluded from the analysis as most schools do not operate school meal programs during summer break.

### Outcome variables

Two outcome measures were used: 1) Average total number of meals served per day, known as Average Daily Participation or ADP, and 2) Average Daily Participation Rate (ADPr). ADP was calculated by dividing the number of meals served during a given time period by the number of school operational days in the same period. ADPr for a specific category (free, reduced-price, paid) was calculated by dividing the ADP for that category by the number of eligible enrolled students in that category and multiplying by 100. An example of the ADP and ADPr calculation for the Free category is provided below:


$$\begin{array}{c} \text{ADP}(\text{Free}):\;\frac{\text{Number of free meals served per month}}{\text{Number of school operational days per month}} \end{array}$$

$$\begin{array}{c} \text{ADPr }(\text{Free}):\;\frac{\text{ADP Free}}{\text{Number of enrolled students eligible for free meals}}*\text{1}\text{0}\text{0} \end{array}$$

Considering both outcomes provides a more nuanced understanding of school meal participation across different school contexts. While ADP is a measure of the total number of meals served, ADPr measures the program’s reach among eligible students.

### Exposure variables

The categorical variable capturing the policy time period (T0-T3) was the key exposure variable. Contextual variables from NCES were included in the analysis to account for compositional differences across schools over time. Locale was categorized as urban (including suburban) or rural. Urban and suburban locations were combined because of the low-density nature of the urban areas and the presence of larger suburban centers in the state. The majority school race variable was derived from the racial/ethnic composition and had four categories: (1) White majority, (2) Hispanic majority, (3) American Indian/Alaskan Native majority (AIAN), and (4) no majority (when no single group was > 50%) and Other majority (Black, Asian and Two or more majority) combined due to low prevalence. Schools were classified based on the lowest and highest grades offered, as elementary, middle, and high schools. Schools were categorized as CEP eligible if their ISP was greater than or equal to 40% in T0-T2, and greater than or equal to 25% in T3.

### Statistical analysis

Analyses were conducted using STATA 18 (SataCorp LLC., College Station, TX). Descriptive statistics were used to examine the distribution of variables included in the analysis. To assess the impact of policy changes on school meal participation (ADP and ADPr), separate mixed effects regression models were run for each eligibility category (free, reduced-price, paid, and for all categories combined) and for each meal type (breakfast and lunch). Consistent with prior research on school meal participation rates [29], mixed-effects models were used to account for the longitudinal structure of the data (i.e., repeated observations nested within schools and schools nested within districts); specifically, the models had 3 levels, and included a random intercept at the school level and one at the district level. All models controlled for school-level factors, such as total enrollment, percentage of students eligible for free and reduced-price meals, locale, majority school race, and school level. Interaction terms between time period and (1) locale, (2) majority school race, and (3) school level were included to assess whether the association between the policy changes and the outcomes varied across these factors. We also considered controlling for a continuous time variable, which would capture a potential secular trend over our study period; however, because time was perfectly correlated with the categorical time period variable (T0–T3), only the categorical variable was included in the models to capture changes in participation associated with policy phases.

After running these multivariable mixed-effects models for both ADP and ADPr, post-estimation analyses were conducted to facilitate interpretation of the results. Predicted ADPr values were generated using the *margins* commands for each eligibility category and meal type. Next, the *lincom* command was used to compare differences in predicted ADPr and ADP across different time periods and school level factors. To estimate the total number of meals served per day on average across all schools in the state for each time period, school-level ADP predictions were generated using the *predict* command and summed across all schools over each study period (T0-T3). Two-sided p-values ≤ 0.05 were considered statistically significant.

### Sensitivity analysis

Because the outcome variable (ADPr) is a proportion, and as such is bounded between 0 and 1, generalized linear models (GLMs) with two-way clustering at the school and district levels were also estimated, using the *vcemway* command, as a robustness check. Sensitivity analyses were also run for ADPr excluding schools participating in CEP at baseline (T0) because these schools would not be impacted by the three policies examined in this analysis. The estimates and the overall conclusions from the analysis did not change. We chose to present results with the full sample of schools as our aim was to present a full picture of school meal participation in the state during the period when new policies were implemented. A summary of findings from the two sensitivity analyses are also presented in the results section.

## Results

### Sample characteristics

A detailed distribution of schools across the four-time periods, by school-level factors is presented in Table 1. The analytical sample comprised 1,730 public and charter schools in Arizona across all time periods. The majority of schools (slightly more than 80%) were located in urban areas. Elementary schools comprised over 70% of the sample, with middle and high schools comprising about 10% and 20%, respectively. About half of the schools had Hispanic majority enrollment across all time periods.

**Table 1:** Distribution of public and charter schools of Arizona across four time periods by total enrollment, school operational days per month, status, locale, school level and majority school race.

	T0 (Aug- Dec 2022)	T1 (Jan- May 2023)	T2 (Aug- Dec 2023)	T3 (Jan- May 2024)
Number of schools (n)	1,705	1,700	1,700	1,699
Total Enrollment	1,020,342	1,006,745	998,477	993,931
Average number of school operational days per month	17	18	17	17
CEP schools (%)				
Yes	26.5	26.8	28.1	43.4
No	73.5	73.2	71.9	56.6
Locale (%)				
Rural	16.4	16.5	16.2	16.8
Urban	81.7	82.2	82.5	82.2
School Level (%)				
Elementary schools	71.5	71.5	71.4	71.6
Middle schools	9.5	9.6	9.8	9.9
High schools	19.0	18.9	18.7	18.5
Majority school race (%)				
White majority	27.3	27.4	27.3	27.5
Hispanic majority	47.0	46.7	47.8	46.8
Black majority	0.1	0.1	0.1	0.1
AIAN majority	4.4	4.6	4.5	4.6
Asian/PI majority	0.2	0.2	0.2	0.2
Two or more/No majority	18.9	19.3	18.5	19.3

**Note:**

T0: Aug- Dec 2022: Post-COVID, three-tiered system in place.

T1: Jan- May 2023: Arizona reduced price co-pay policy implemented.

T2: Aug- Dec 2023: Arizona policy with Medicaid Direct Certification.

T3: Jan- May 2024: Arizona policy with Medicaid Direct Certification and CEP expansion/mid-year enrollment.

### Trends in ADPr across four time periods

#### For all schools.

**Fig 2:**
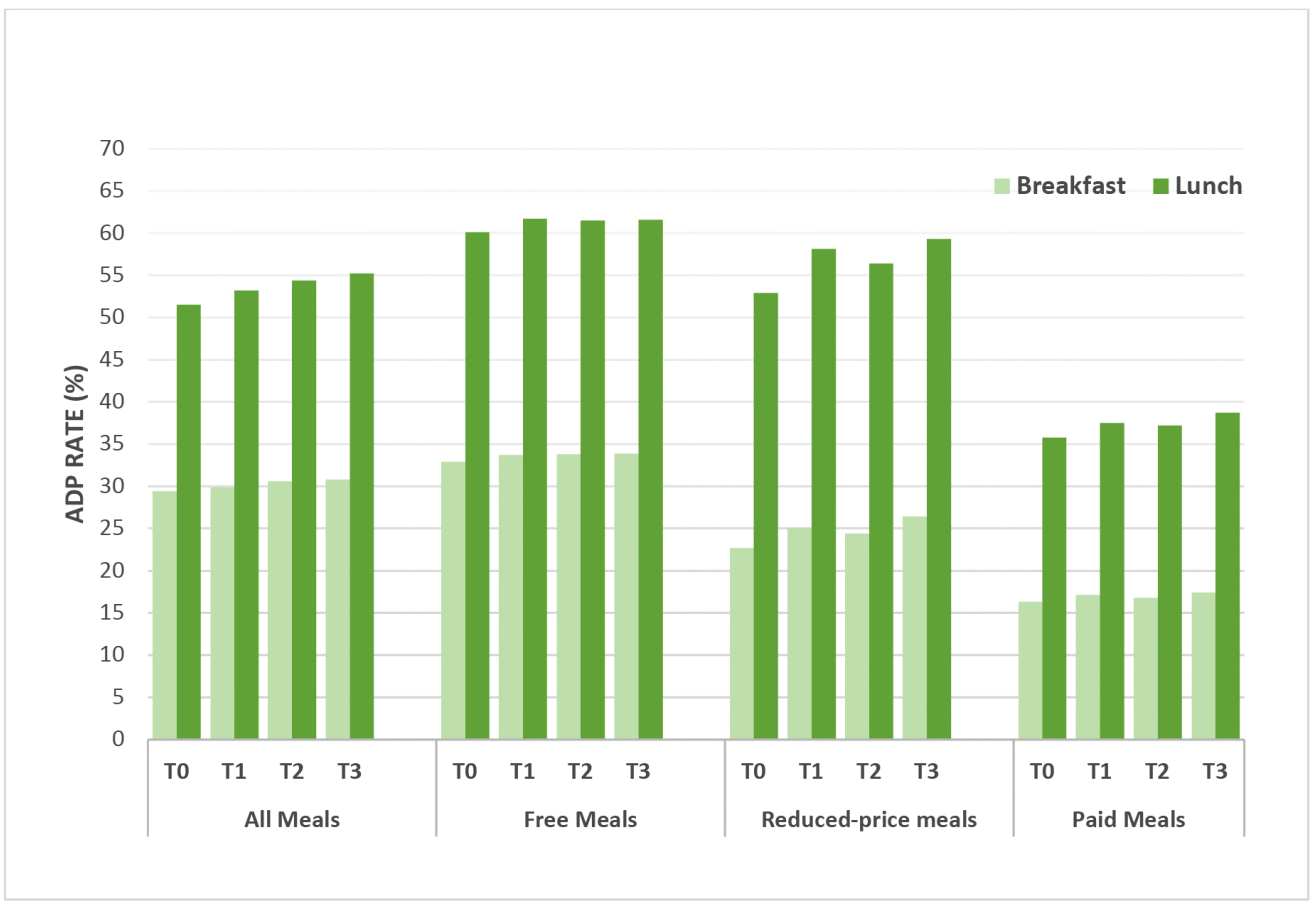
Adjusted average daily participation rate (ADPr) for breakfast and lunch across four policy time periods. Note: T0: Aug- Dec 2022: Post-COVID, three-tiered system in place. T1: Jan- May 2023: Arizona reduced price co-pay policy implemented. T2: Aug- Dec 2023: Arizona policy with Medicaid Direct Certification. T3: Jan- May 2024: Arizona policy with Medicaid Direct Certification and CEP expansion/mid-year enrollment.

Fig 2 presents adjusted ADPr across all time periods. Following the implementation of Arizona’s reduced-price co-pay policy, ADPr for reduced-price breakfast increased by 10% from 22.7% at T0 to 25.0% at T1 (p < 0.001). After the implementation of all three policies, ADPr for breakfast, across all categories, increased by 4.8% from 29.4% at T0 to 30.8% at T3 (p < 0.001). ADPr for free breakfast increased by 3.0% from 32.9% at T0 to 33.9% at T3 (p < 0.001).

As shown in Fig 2, the implementation of Arizona’s reduced-price co-pay policy led to an increase in ADPr for reduced-price lunch by 10%, from 52.9% at T0 to 58.1% at T1 (p < 0.001). Following the implementation of all three policies, ADPr for lunch across all categories increased by 7.2% from 51.4% in T0 to 55.2% in T3 (p < 0.001). ADPr for free lunch showed a small increase, changing from 60.1% in T0 to 61.6% at T3 (p < 0.001).

#### By school level factors.

ADPr for breakfast and lunch across all eligibility categories was consistently higher in rural schools, elementary schools, and Hispanic and AIAN majority schools across all time periods. After the implementation of state and federal policies (in T3) middle schools saw a significantly greater increase in breakfast participation compared to elementary (p = 0.006) and high schools (p = 0.003). Similarly, Hispanic majority schools saw a significantly greater increase in breakfast participation compared to White (p < 0.001) and AIAN majority schools (p < 0.001). For lunch, following the implementation of all three policies (in T3) urban schools saw a significantly greater increase compared to rural schools (p < 0.001); elementary schools saw a significantly larger increase compared to middle (p = 0.018) and high schools (p < 0.001); and White majority schools saw a significantly larger increase compared to Hispanic, AIAN or no majority schools (p < 0.001, for all). A detailed report of the ADPr across all time periods, eligibility categories and school level factors are provided in Table 2 and in S1 and S2 Tables [30].

**Table 2:** Adjusted average daily participation rate (ADPr) for breakfast and lunch across all eligibility category and across four time periods (Mean, 95% CI).

ADPr for breakfast across all eligibility category											
	T0		T1			T2			T3		
	(n = 1,705)		(n = 1,700)			(n = 1,700)			(n = 1,699)		
	Mean	95% CI	Mean	95% CI	p-value (T1-T0)	Mean	95% CI	p-value (T2-T0)	Mean	95% CI	p-value (T3-T0)
**Overall**	29.4	27.6–31.3	29.9	28.0–31.8	**<0.001**	30.6	28.7–32.5	**<0.001**	30.8	28.9–32.7	**<0.001**
**Locale**											
Rural	34.2	31.7–36.7	35.1	32.6–37.6	**<0.001**	36.1	33.6–38.6	**<0.001**	35.2	32.7–37.8	**<0.001**
Urban	28.5	26.5–30.5	28.9	26.9–30.8	**<0.001**	29.5	27.5–31.5	**<0.001**	29.9	27.9–31.9	**<0.001**
**School Level**											
Elementary	32.5	30.6–34.5	33.2	31.2–35.1	**<0.001**	33.7	31.7–35.7	**<0.001**	33.8	31.9–35.8	**<0.001**
Middle	21.7	18.9–24.4	21.9	19.1–24.7	0.419	23.0	20.3–25.8	**<0.001**	23.8	21.0–26.6	**<0.001**
High	21.9	19.5–24.3	21.7	19.4–24.1	0.486	22.9	20.5–25.3	**<0.001**	23.0	20.6–25.3	**<0.001**
**Majority school race**											
White majority	18.2	15.8–20.7	19.2	16.8–21.7	**<0.001**	19.0	16.5–21.5	**<0.001**	19.6	17.2–22.1	**<0.001**
Hispanic majority	35.7	33.6–37.7	36.2	34.1–38.3	**<0.001**	37.4	35.4–39.5	**<0.001**	37.5	35.4–39.6	**<0.001**
AIAN majority	41.3	34.8–47.7	36.1	29.6–42.5	**<0.001**	39.3	32.9–45.8	**<0.001**	37.0	30.5–43.4	**<0.001**
No majority	26.7	24.2–29.2	27.6	25.1–30.1	**<0.001**	27.6	25.1–30.1	**<0.001**	28.1	25.6–30.6	**<0.001**
**ADPr for lunch across all eligibility category**											
	**T0**		**T1**			**T2**			**T3**		
	**Mean**	**95% CI**	**Mean**	**95% CI**	**p-value (T1-T0)**	**Mean**	**95% CI**	**p-value (T2-T0)**	**Mean**	**95% CI**	**p-value (T3-T0)**
**Overall**	51.4	50.2–52.8	53.2	51.9–54.5	**<0.001**	54.4	53.1–55.7	**<0.001**	55.2	53.9–56.5	**<0.001**
**Locale**											
Rural	54.2	52.3–56.0	55.8	53.9–57.7	**<0.001**	56.7	54.8–58.6	**<0.001**	57.2	55.3–59.1	**<0.001**
Urban	51.0	49.6–52.4	52.7	51.3–54.1	**<0.001**	54.0	52.6–55.4	**<0.001**	54.8	53.4–56.2	**<0.001**
**School Level**											
Elementary	54.6	53.2–56.0	57.2	55.8–58.6	**<0.001**	57.5	56.1–58.9	**<0.001**	58.8	57.4–60.2	**<0.001**
Middle	48.0	45.9–50.1	47.7	45.7–49.8	0.41	51.5	49.4–53.6	**<0.001**	51.1	49.1–53.2	**<0.001**
High	41.6	39.9–43.3	40.8	39.1–42.5	**<0.001**	44.3	42.6–46.1	**<0.001**	43.7	41.9–45.4	**<0.001**
**Majority school race**											
White majority	41.3	39.5–43.1	43.8	42.0–45.6	**<0.001**	43.8	42.0–45.6	**<0.001**	45.4	43.6–47.2	**<0.001**
Hispanic majority	57.8	56.3–59.3	59.0	57.4–60.5	**<0.001**	60.7	59.2–62.2	**<0.001**	61.3	59.8–62.8	**<0.001**
AIAN majority	61.2	56.4–66.0	60.3	55.5–65.0	**0.036**	64.1	59.3–68.8	**<0.001**	62.4	57.6–67.2	**0.008**
No majority	48.2	46.4–50.1	50.7	48.9–52.6	**<0.001**	51.9	50.1–53.7	**<0.001**	52.8	51.0–54.6	**<0.001**

**Note:**

T0: Aug- Dec 2022: Post-COVID, three-tiered system in place.

T1: Jan- May 2023: Arizona reduced price co-pay policy implemented.

T2: Aug- Dec 2023: Arizona policy with Medicaid Direct Certification.

T3: Jan- May 2024: Arizona policy with Medicaid Direct Certification and CEP expansion/mid-year enrollment.

### Trends in ADP across four time periods

#### For all schools.

**Fig 3:**
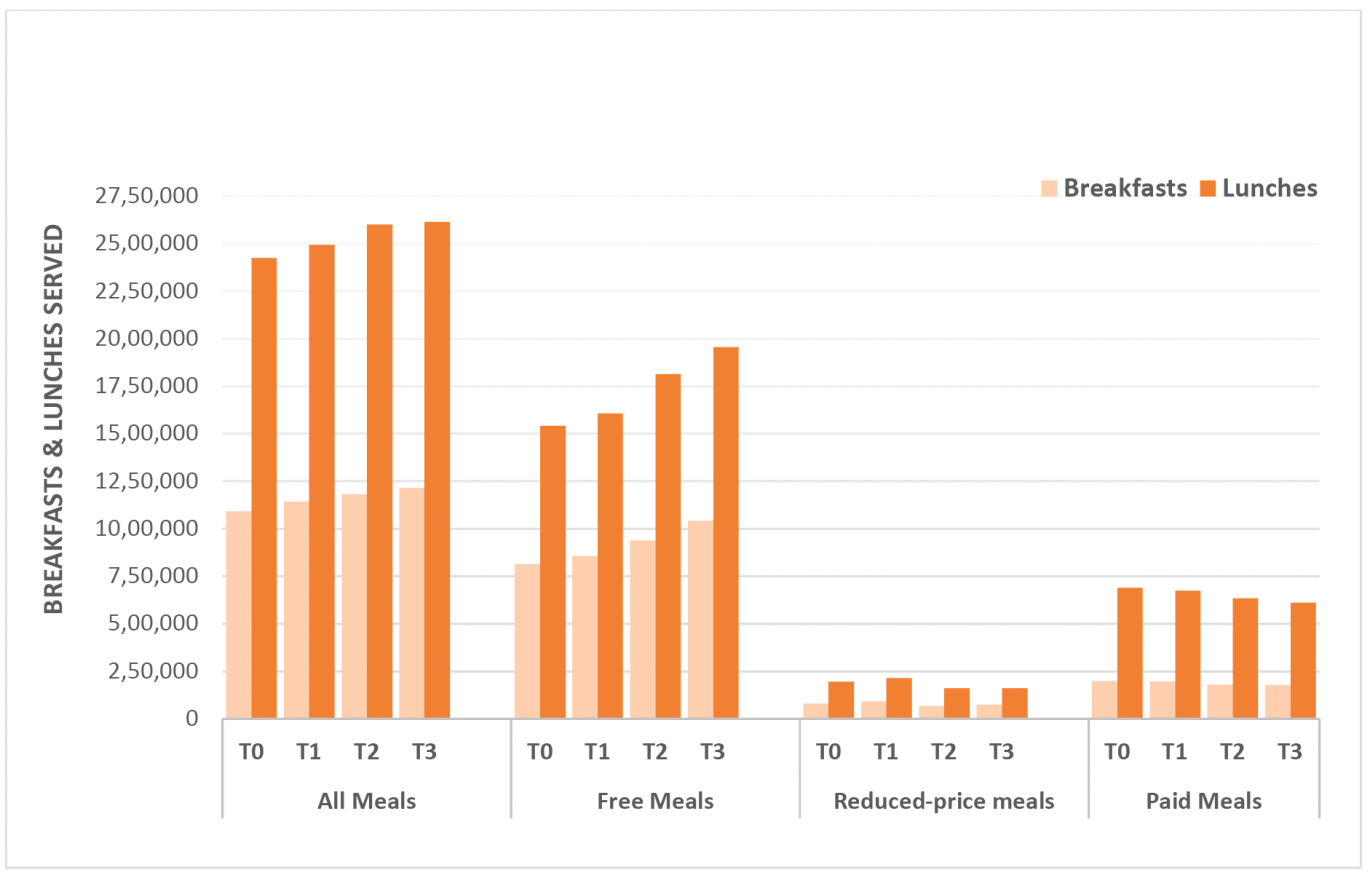
Total number of breakfasts and lunches served each day on average across four policy time periods. Note: T0: Aug- Dec 2022: Post-COVID, three-tiered system in place. T1: Jan- May 2023: Arizona reduced price co-pay policy implemented. T2: Aug- Dec 2023: Arizona policy with Medicaid Direct Certification. T3: Jan- May 2024: Arizona policy with Medicaid Direct Certification and CEP expansion/mid-year enrollment.

Fig 3 presents adjusted ADP across all time periods for all Arizona schools. The number of breakfasts served to Arizona students per day on average increased by 121,897 (11.2%) at T3 from a baseline of 1,092,617 at T0. The largest gain was observed in free breakfasts, which increased by 227,190 meals per day at T3, a 27.9% increase from 814,853 at T0. At T1, reduced-price breakfasts increased by 10,136 meals (12.5%) from 80,341 in T0, but declined in subsequent periods as more students became eligible for free meals. In contrast, paid breakfasts saw a steady decline over the time periods, and by T3, 22,105 fewer paid meals were served per day, from an initial value of 198,891 in T0.

Post implementation of all three policies, the total number of lunches served daily increased by 189,292 meals (7.8%) in T3 from a baseline of 2,424,444 in T0 (Fig 3). Free lunches saw the largest increase, up by 413,886 meals per day at T3, a 26.8% rise from the initial 1,542,055 lunches at T0. Reduced-price lunches initially rose by 17,552 meals (9.2%) from 196,050 in T0 to T1, but declined in later periods due to expanded eligibility for free meals. Paid lunches declined by 78,602 meals at T3 from an initial value of 689,192 at T0.

#### By school level factors.

The total number of breakfasts and lunches served daily on average across all eligibility categories was consistently higher in urban schools, elementary schools, and Hispanic majority schools across all time periods.

Following the implementation of state and federal policies, urban schools (compared to rural schools, p < 0.001), elementary and high schools (compared to middle schools, p < 0.001, for both), and Hispanic majority schools (compared to White, AIAN, and no-Majority schools, p < 0.001, for all) saw greater increases in the average number of breakfasts served daily across all eligibility categories. A similar pattern was observed for the average number of lunches served daily across all eligibility categories.

A detailed report of the average number of breakfasts and lunches served per day across all time periods by eligibility categories and school-level factors is provided in Table 3 and in S3 and S4 Tables [30].

**Table 3:** Total number of breakfasts and lunches served daily on average across all eligibility categories and all time periods (Total, SE).

Total number of breakfasts served daily on average across all eligibility category											
	T0		T1			T2			T3		
	(n = 1,705)		(n = 1,700)			(n = 1,700)			(n = 1,699)		
	Total	SE	Total	SE	p-value (T1-T0)	Total	SE	p-value (T2-T0)	Total	SE	p-value (T3-T0)
**Overall**	1,092,617	12,866	1,141,664	12,899	**0.007**	1,182,616	12,928	**<0.001**	1,214,514	12,974	**<0.001**
**Locale**											
Rural	143,020	4,223	146,382	4,208	0.573	159,131	4,274	**0.007**	153,818	4,207	0.07
Urban	949,597	12,096	995,282	12,125	**0.008**	1,023,485	12,140	**<0.001**	1,060,696	12,194	**<0.001**
**School Level**											
Elementary	860,042	10,473	894,298	10,477	**0.021**	904,172	10,574	**0.003**	923,766	10,633	**<0.001**
Middle	83,455	2,975	86,827	2,991	0.424	87,815	3,006	0.303	92,967	3,040	**0.025**
High	149,120	6,566	160,539	6,603	0.22	190,628	6,631	**<0.001**	197,781	6,634	**<0.001**
**Majority school race**											
White majority	128,454	3,108	141,224	3,105	**0.004**	145,988	3,093	**<0.001**	151,071	3,072	**<0.001**
Hispanic majority	763,467	10,551	793,715	10,671	**0.044**	815,636	10,525	**<0.001**	846,328	10,575	**<0.001**
AIAN majority	47,886	1,870	44,292	1,875	0.175	49,651	1,886	0.506	45,953	1,825	0.46
No majority	152,811	3,393	162,433	3,372	**0.044**	171,341	3,421	**<0.001**	171,162	3,334	**<0.001**
**Total number of lunches served daily on average across all eligibility category**											
	**T0**		**T1**			**T2**			**T3**		
	**Total**	**SE**	**Total**	**SE**	**p-value (T1-T0)**	**Total**	**SE**	**p-value (T2-T0)**	**Total**	**SE**	**p-value (T3-T0)**
**Overall**	2,424,444	18,461	2,493,595	18,489	**0.008**	2,601,262	18,749	**<0.001**	2,613,736	18,655	**<0.001**
**Locale**											
Rural	287,826	6,735	295,658	6,784	0.413	316,311	6,897	**0.003**	307,876	6,826	**0.037**
Urban	2,136,617	16,853	2,197,937	16,852	**0.01**	2,284,951	17,063	**<0.001**	2,305,860	16,980	**<0.001**
**School Level**											
Elementary	1,702,094	12,214	1,783,830	12,221	**<0.001**	1,791,731	12,316	**<0.001**	1,830,516	12,311	**<0.001**
Middle	217,834	4,234	217,861	4,244	0.996	234,721	4,363	**0.005**	227,796	4,327	0.1
High	504,516	13,111	491,904	13,184	0.498	574,810	13,258	**<0.001**	555,424	13,221	**0.006**
**Majority school race**											
White majority	544,110	7,677	566,865	7,605	**0.035**	582,188	7,810	**0.001**	584,435	7,684	**<0.001**
Hispanic majority	1,378,436	14,543	1,413,585	14,705	0.089	1,462,954	14,685	**<0.001**	1,493,259	14,726	**<0.001**
AIAN majority	76,475	2,624	75,096	2,745	0.717	84,025	2,790	**0.049**	78,331	2,744	0.625
No majority	425,422	6,156	438,049	6,169	0.147	472,096	6,292	**<0.001**	457,712	6,182	**<0.001**

**Note:**

T0: Aug- Dec 2022: Post-COVID, three-tiered system in place.

T1: Jan- May 2023: Arizona reduced price co-pay policy implemented.

T2: Aug- Dec 2023: Arizona policy with Medicaid Direct Certification.

T3: Jan- May 2024: Arizona policy with Medicaid Direct Certification and CEP expansion/mid-year enrollment.

### Trends in CEP school participation

As shown in Table 4, prior to the CEP policy change, 62.9% (n = 451) of eligible schools participated in CEP at T0, 63.3% (n = 456) at T1, and 40.6% (n = 477) at T2. Following the expansion of the USDA eligibility threshold at T3, participation in CEP among eligible schools was 52.2% (n = 737). This expansion enabled an additional 147,125 students in Arizona to access free school meals between T2 and T3. Overall, the ADPr in CEP schools slightly decreased after the CEP post-policy change. While the total number of both urban and rural schools participating in CEP increased from T0-T2 to T3, the percentage of eligible schools participating in CEP decreased from 80.3% (n = 98) to 47.9% (n = 112) for rural schools and from 59.0% (n = 343) to 52.7% (n = 613) for urban schools from baseline to T3 as more schools became eligible. A similar trend was observed by school-level; between T0 and T3, the share of eligible schools participating in CEP decreased from 61.6% (n = 330) to 54.9% (n = 557), from 66.7% (n = 46) to 52.9% (n = 72), and from 66.7% (n = 74) to 41.2% (n = 107), for elementary, middle, and high schools, respectively.

**Table 4:** CEP participation among eligible schools and students’ access to, and participation in, free meals across four policy time periods.

Total number of schools eligible for CEP overall and by locale and school level												
	T0 (Aug-Dec 2022)			T1 (Jan-May 2023)			T2 (Aug-Dec 2023)			T3 (Jan-May 2024)		
	CEP eligible schools	Number of CEP schools	% schools	CEP eligible schools	Number of CEP schools	% schools	CEP eligible schools	Number of CEP schools	% schools	CEP eligible schools	Number of CEP schools	% schools
	N	N	%	N	N	%	N	N	%	N	N	%
**Overall**	717	451	62.9	720	456	63.3	1174	477	40.6	1413	737	52.2
**Locale**												
Rural	122	98	80.3	124	100	80.6	190	101	53.2	234	112	47.9
Urban	581	343	59.0	582	346	59.5	968	365	37.7	1164	613	52.7
**School Level**												
Elementary	536	330	61.6	539	334	62.0	846	351	41.5	1016	557	54.8
Middle	69	46	66.7	69	46	66.7	117	49	41.9	136	72	52.9
High	111	74	66.7	111	75	67.6	210	76	36.2	260	107	41.2
**Number of enrolled students and average daily participation in CEP schools overall and by locale and school level**												
	**T0 (Aug-Dec 2022)**			**T1 (Jan-May 2023)**			**T2 (Aug-Dec 2023)**			**T3 (Jan-May 2024)**		
	**Total enrolled students**	**Average daily participation (ADPr)**		**Total enrolled students**	**Average daily participation (ADPr)**		**Total enrolled students**	**Average daily participation (ADPr)**		**Total enrolled students**	**Average daily participation (ADPr)**	
	**N**	**Mean**	**SD**	**N**	**Mean**	**SD**	**N**	**Mean**	**SD**	**N**	**Mean**	**SD**
**Overall**	173,601	69.2	14.5	172,366	69.2	15.4	179,771	72.1	14.6	326,896	68.3	15.5
**Locale**												
Rural	26,403	71.6	13.6	26,713	71.2	14.2	26,794	73.5	14.3	294,368	71.4	13.6
Urban	146,316	68.3	14.7	144,775	68.3	15.7	151,995	71.3	14.7	31,456	67.5	15.7
**School Level**												
Elementary	124,557	73.6	10.2	125,066	74.6	10.0	130,536	76.6	9.5	237,321	72.8	11.4
Middle	21,796	66.8	15.7	20,879	63.7	15.2	21,814	69.9	13.5	33,811	64.3	13.0
High	27,061	51.6	16.7	26,234	48.5	16.9	27,227	52.4	18.1	55,568	47.7	18.2

**Note:**

T0: Aug- Dec 2022: Post-COVID, three-tiered system in place.

T1: Jan- May 2023: Arizona reduced price co-pay policy implemented.

T2: Aug- Dec 2023: Arizona policy with Medicaid Direct Certification.

T3: Jan- May 2024: Arizona policy with Medicaid Direct Certification and CEP expansion/mid-year enrollment.

*As few schools had missing values for School Level, Locale, and Majority School Race in the NCES data, the column totals for these categories may not sum to the total schools.

*CEP eligibility defined as CEP participating schools or those with an ISP greater than or equal to 40% for T0-T2 prior to federal expansion policy, and participating CEP schools or those with an ISP greater than or equal to 25% for T3 after federal expansion policy.

### Sensitivity analysis

In the first sensitivity analysis, we ran the analysis using generalized linear models (GLMs) with two-way clustering at the school and district levels, using the *vcemway* command, as a robustness check. For breakfast, reduced models had to be estimated due to non-convergence of the full model. For each outcome, the results obtained with *glm* were entirely consistent, supporting the stability of the findings, and validating the use of mixed-effects linear models.

In the second sensitivity analysis we excluded schools that were participating in CEP at baseline. As expected, the overall ADPr for breakfast and lunch was lower in the sensitivity analysis sample, given there were fewer schools in the sample that offered free meals to all students. Lunch participation in the sensitivity analysis increased from 46.3% to 50.1% (a 3.8% point increase) between T0 and T3 compared to the full sample where it changed from 51.4% to 55.2% (a 3.8% point increase). Similarly, breakfast participation in the sensitivity analysis increased by 2% points compared to 1.4% point increase in the full sample.

## Discussion

This study assessed the changes in school meal participation across a period (August 2022 to May 2024) during which one state-level and two federal-level policies were implemented sequentially in the state of Arizona. We assess school meal participation using two outcomes ADP and ADPr, ADP captures the total number of meals served, and ADPr measures the program’s reach among eligible students. We found that ADPr for both lunch and breakfast increased significantly across eligibility categories compared to baseline. Specifically, the elimination of reduced-price meal co-pay led to an immediate 10% increase in ADPr among students eligible for reduced-price meals for both breakfast and lunch. By removing this financial barrier, Arizona’s policy expanded access for low-income students who were previously not eligible for free meals. Prior studies have identified meal cost as a key reason for nonparticipation in school meals [31], and a recent USDA study has shown that nearly one-third of households paying for school meals reported difficulty affording other usual expenses [32]. The Arizona policy likely provided meaningful relief to such families, contributing to higher participation.

Implementation of Medicaid Direct Certification further improved meal access by enabling automatic certification of students in Medicaid households and reducing administrative burdens for families and schools [33]. This policy also indirectly supported greater adoption of CEP, as more students were directly certified, leading to higher school ISPs. Consistent with this mechanism, the number of CEP-eligible schools increased from 720 at T1 to 1,174 at T2. This increase in the number of schools eligible for CEP at T2 also contributed to the lower rate of CEP participation during this period, as schools could enroll in CEP until January 2024 (T3).

Although the expansion of CEP eligibility in T3 increased the total number of schools participating in CEP to 737, the proportion of eligible schools enrolled in CEP decreased after policy implementation. This pattern likely reflects the financial challenges faced by schools with lower ISPs, for which current USDA reimbursement rates do not fully offset costs [15]. This can be seen in our sample, where at T3, 70.6% of schools with ISP above 60% participated in CEP, compared to 39.3% of the eligible schools between 25–60% threshold. These findings underscore a persistent limitation of the CEP reimbursement formula, which may deter participation among lower-ISP schools even after eligibility expands.

While we saw an increase in breakfast and lunch participation after policy implementation, consistent with prior research, participation in breakfast continued to lag behind participation in school lunches [2,34]. This differential suggests that while state and federal policies are critical for increasing access, school-level operational factors- such as breakfast delivery models can also play an important role. Implementation of alternative strategies, such as breakfast after the bell and second chance breakfast, have been shown to improve participation [35] and should be scaled up as breakfast participation is associated with positive dietary and academic outcomes [36–38]. The observed decline in reduced-price and paid meal participation at T2 and T3 is likely due to more students qualifying for free meals through Medicaid Direct Certification and expanded CEP eligibility. In this scenario, it is likely that the students remaining in the reduced-price category attended higher income schools, where meal participation is typically lower. The combined positive impact of all three policies is evident in the 27.9% and 26.8% increases in the average daily number of free breakfasts and lunches served, respectively. Together, these policies expanded meal access by addressing long-standing barriers in the traditional 3-tiered framework, including stigma and application burden [14].

Participation increased across all school level subgroups after implementation of all three policies. Elementary schools had consistently higher participation rates across all eligibility categories compared to middle and high schools, consistent with previous studies showing younger students are more likely to participate in school meal programs [31,39]. Hispanic and AIAN majority schools also had higher participation rates, similar to the findings from the School Nutrition and Meal Cost Study (SNMCS) [39]. Similar to SNMCS [39], rural schools in our sample had higher participation rate than urban schools, likely due to higher poverty levels in rural areas [40]. However, urban schools showed greater gains in lunch participation, following the implementation of all three policies, specifically the CEP expansion, as more urban schools enrolled in CEP after the policy change providing free meal to all enrolled students.

To conclude, the three sequential policy interventions substantially improved access to free school meals for students in Arizona, highlighting the potential for alignment between state and federal policies to enhance access to school meals. Expanding access to school meals is a crucial public health strategy, as extensive research links school meal participation to improved food security, dietary quality, academic performance, and overall child well-being [14]. Thus, policies that increase participation have important implications for reducing food insecurity and supporting better health outcomes among children.

### Strengths and limitations

This study has several strengths. It utilized longitudinal administrative data covering all public and charter schools in Arizona, providing comprehensive and accurate estimates of school meal participation. By examining overall trends as well as differences across meal eligibility categories and school-level characteristics, the analysis offers important insights into the equity of impacts of these policies. Finally, to our knowledge, this is among the first studies to assess the combined impact of concurrent state and federal policy changes on school meal participation.

However, findings may not be generalizable to other states with different demographic or policy contexts. The absence of a traditional control group is another limitation; nonetheless, the pre-implementation period provided a reasonable baseline for assessing changes in participation over time. Our study was descriptive in nature, limited to a single state, focused on short-term participation changes during a relatively compressed sequence of policy reforms. Future research should examine long-term impacts of these policy changes on participation, academic performance, weight status, and food insecurity. Finally, despite adjusting for several key school-level covariates, unmeasured confounding factors, such as local implementation practices or cafeteria-level dynamics, may have also influenced meal participation.

## Supporting information

S1 Table Adjusted average daily participation rate (ADPr) – Breakfast. (DOCX)

S2 Table Adjusted average daily participation rate (ADPr) – Lunch. (DOCX)

S3 Table Total number of breakfasts served daily (adjusted). (DOCX)

S4 Table Total number of lunches served daily (adjusted). (DOCX)
