# Supplementary material for: State and federal policies and school meal participation: A descriptive analysis from Arizona
Source: PLoS One. 2026 Jun 12;21(6):e0350416. doi: 10.1371/journal.pone.0350416 (PMC13262862; doi:10.1371/journal.pone.0350416)
Supplement: S2 Table — (DOCX) [file pone.0350416.s002.docx]

**S2 Table. Adjusted Average Daily Participation Rate (ADPr) – Lunch**

**A.** ADPr for free lunch across four policy time periods (Means, 95% CI)

**B.** ADPr for reduced-price lunch across four policy time periods (Means, 95% CI)

**C.** ADPr for paid lunch across four policy time periods (Means, 95% CI)

The tables below show results derived from regression models analyzing ADPr for lunch across multiple policy time periods (T0–T3), and eligibility categories.

***Model specification:*** Separate mixed-effects models with robust standard errors were run for each eligibility category (free, reduced-price, and paid). Each model controlled for school-level factors such as total enrollment and percentage of students eligible for free and reduced-price meals. Interaction terms between time period and locale, majority school race, and school level were included in the models to assess whether the effect of policy changes varied across these factors.

***Clustering:*** Standard errors were clustered at both the school and district level to account for within-group correlation.

***Dependent variables:*** ADPr for lunch, for each eligibility category.

***ADPr outcomes:*** After estimating models, post-estimation margins were used to obtain adjusted means and 95% confidence intervals. Pairwise differences between time periods (T1, T2, T3) and T0 were tested using `*lincom*’.

***Independent variables:*** Time period (T0, T1, T2, T3) and school-level factors, including school level, locale, majority school race, student enrollment, and proportion of students eligible for free and reduced-price meals. No transformations were applied to these variables.

***Observations:*** The number of observations differed based on eligibility category.

***Reported estimates:*** For each model, estimated means and 95% confidence intervals (CIs) or standard errors (SEs) are presented across policy periods, along with p-values testing differences from the baseline (T0).

*Regression outputs (coefficients, standard errors, R-squared, F-statistics) are available upon request.

**A.** Adjusted Average Daily Participation Rate (ADPr) for free lunch across four policy time period (Means, 95% CI)

|  | **T0** | | **T1** | | | **T2** | | | **T3** | | |
| --- | --- | --- | --- | --- | --- | --- | --- | --- | --- | --- | --- |
|  | **(n=1,705)** | | **(n=1,700)** | | | **(n=1,700)** | | | **(n=1,699)** | | |
|  | **Mean** | **95% CI** | **Mean** | **95% CI** | **p-value (T1-T0)** | **Mean** | **95% CI** | **p-value (T2-T0)** | **Mean** | **95% CI** | **p-value (T3-T0)** |
| **Overall** | 60.1 | 58.9–61.3 | 61.7 | 60.5–62.9 | **<0.001** | 61.5 | 60.3–62.7 | **<0.001** | 61.6 | 60.4–62.8 | **<0.001** |
| **Locale** |  |  |  |  |  |  |  |  |  |  |  |
| Rural | 61.8 | 60.0–63.5 | 63.3 | 61.6–65.1 | **<0.001** | 63.4 | 61.6–65.1 | **<0.001** | 63.5 | 61.8–65.3 | **<0.001** |
| Urban | 59.8 | 58.5–61.1 | 61.4 | 60.1–62.7 | **<0.001** | 61.1 | 59.8–62.4 | **<0.001** | 61.3 | 60.0–62.5 | **<0.001** |
| **School Level** |  |  |  |  |  |  |  |  |  |  |  |
| Elementary | 63.8 | 62.6–65.1 | 66.2 | 64.9–67.5 | **<0.001** | 65.1 | 63.9–66.4 | **<0.001** | 65.7 | 64.4–66.9 | **<0.001** |
| Middle | 55.6 | 53.6–57.6 | 55.4 | 53.4–57.4 | 0.528 | 57.6 | 55.6–59.6 | **<0.001** | 56.9 | 54.9–58.9 | **<0.001** |
| High | 48.5 | 46.9–50.2 | 47.9 | 46.3–49.5 | **0.007** | 49.7 | 48.0–51.3 | **<0.001** | 48.8 | 47.1–50.4 | 0.35 |
| **Majority school race** |  |  |  |  |  |  |  |  |  |  |  |
| White majority | 54.4 | 52.7–56.0 | 57 | 55.3–58.7 | **<0.001** | 53.6 | 51.9–55.2 | **<0.001** | 55.1 | 53.4–56.7 | **<0.001** |
| Hispanic majority | 63.9 | 62.5–65.3 | 64.8 | 63.4–66.1 | **<0.001** | 66.1 | 64.7–67.4 | **<0.001** | 65.8 | 64.4–67.1 | **<0.001** |
| AIAN majority | 65.9 | 61.5–70.2 | 64.8 | 60.5–69.2 | **0.041** | 68.4 | 64.1–72.7 | **<0.001** | 66.5 | 62.1–70.8 | 0.226 |
| No majority | 57.9 | 56.1–59.6 | 60.3 | 58.6–62.0 | **<0.001** | 59.9 | 58.2–61.6 | **<0.001** | 59.8 | 58.1–61.5 | **<0.001** |

**B.** Adjusted Average Daily Participation Rate (ADPr) for reduced-price lunch across four policy time period (Means, 95% CI)

|  | **T0** | | **T1** | | | **T2** | | | **T3** | | |
| --- | --- | --- | --- | --- | --- | --- | --- | --- | --- | --- | --- |
|  | **(n=1,705)** | | **(n=1,700)** | | | **(n=1,700)** | | | **(n=1,699)** | | |
|  | **Mean** | **95% CI** | **Mean** | **95% CI** | **p-value (T1-T0)** | **Mean** | **95% CI** | **p-value (T2-T0)** | **Mean** | **95% CI** | **p-value (T3-T0)** |
| **Overall** | 52.9 | 51.0–54.7 | 58.1 | 56.3–59.9 | **<0.001** | 56.4 | 54.6–58.3 | **<0.001** | 59.3 | 57.5–61.1 | **<0.001** |
| **Locale** |  |  |  |  |  |  |  |  |  |  |  |
| Rural | 54.7 | 52.3–57.1 | 59.4 | 56.9–61.8 | **<0.001** | 57.4 | 55.0–59.9 | **<0.001** | 60.1 | 57.6–62.5 | **<0.001** |
| Urban | 52.6 | 50.7–54.4 | 57.9 | 56.0–59.8 | **<0.001** | 56.3 | 54.4–58.1 | **<0.001** | 59.2 | 57.3–61.1 | **<0.001** |
| **School Level** |  |  |  |  |  |  |  |  |  |  |  |
| Elementary | 56.8 | 54.9–58.7 | 62.9 | 61.0–64.8 | **<0.001** | 59.5 | 57.6–61.4 | **<0.001** | 62.9 | 61.0–64.8 | **<0.001** |
| Middle | 47.3 | 44.7–49.9 | 50.3 | 47.7–52.9 | **<0.001** | 53.3 | 50.7–55.9 | **<0.001** | 55.2 | 52.6–57.9 | **<0.001** |
| High | 41.2 | 39.0–43.5 | 44.3 | 42.1–46.6 | **<0.001** | 46.7 | 44.5–49.0 | **<0.001** | 48 | 45.8–50.3 | **<0.001** |
| **Majority school race** |  |  |  |  |  |  |  |  |  |  |  |
| White majority | 50.6 | 48.4–52.7 | 56.1 | 54.0–58.2 | **<0.001** | 53.5 | 51.3–55.6 | **<0.001** | 56.8 | 54.7–59.0 | **<0.001** |
| Hispanic majority | 55 | 53.0–57.0 | 59.8 | 57.8–61.8 | **<0.001** | 59.5 | 57.5–61.5 | **<0.001** | 62.3 | 60.3–64.3 | **<0.001** |
| AIAN majority | - | - | - | - | **-** | - | - | **-** | - | - | **-** |
| No majority | 52.3 | 50.1–54.6 | 58 | 55.8–60.3 | **<0.001** | 55.4 | 53.1–57.6 | **<0.001** | 57.6 | 55.3–59.8 | **<0.001** |

**C.** Adjusted Average Daily Participation Rate (ADPr) for paid lunch across four policy time period (Means, 95% CI)

|  | **T0** | | **T1** | | | **T2** | | | **T3** | | |
| --- | --- | --- | --- | --- | --- | --- | --- | --- | --- | --- | --- |
|  | **(n=1,705)** | | **(n=1,700)** | | | **(n=1,700)** | | | **(n=1,699)** | | |
|  | **Mean** | **95% CI** | **Mean** | **95% CI** | **p-value (T1-T0)** | **Mean** | **95% CI** | **p-value (T2-T0)** | **Mean** | **95% CI** | **p-value (T3-T0)** |
| **Overall** | 35.8 | 33.6–37.9 | 37.5 | 35.4–39.6 | **<0.001** | 37.2 | 35.1–39.3 | **<0.001** | 38.7 | 36.6–40.8 | **<0.001** |
| **Locale** |  |  |  |  |  |  |  |  |  |  |  |
| Rural | 37.9 | 35.3–40.4 | 39.5 | 37.0–42.0 | **<0.001** | 38.1 | 35.6–40.7 | 0.362 | 39.4 | 36.9–41.9 | **<0.001** |
| Urban | 35.4 | 33.2–37.5 | 37.2 | 35.0–39.3 | **<0.001** | 37 | 34.9–39.1 | **<0.001** | 38.6 | 36.4–40.7 | **<0.001** |
| **School Level** |  |  |  |  |  |  |  |  |  |  |  |
| Elementary | 38.2 | 36.1–40.4 | 40.9 | 38.7–43.0 | **<0.001** | 39.2 | 37.0–41.3 | **<0.001** | 41.4 | 39.2–43.6 | **<0.001** |
| Middle | 33.8 | 31.2–36.5 | 34.3 | 31.7–36.9 | 0.196 | 37.1 | 34.5–39.8 | **<0.001** | 36.9 | 34.3–39.5 | **<0.001** |
| High | 27.9 | 25.6–30.3 | 27.3 | 25.0–29.7 | **0.02** | 30.1 | 27.7–32.5 | **<0.001** | 29.9 | 27.6–32.3 | **<0.001** |
| **Majority school race** |  |  |  |  |  |  |  |  |  |  |  |
| White majority | 32.1 | 29.8–34.4 | 34.4 | 32.1–36.7 | **<0.001** | 33.8 | 31.4–36.1 | **<0.001** | 35 | 32.7–37.3 | **<0.001** |
| Hispanic majority | 39.7 | 37.4–42.0 | 40.8 | 38.6–43.1 | **<0.001** | 40.5 | 38.3–42.8 | **<0.001** | 42.8 | 40.5–45.0 | **<0.001** |
| AIAN majority | - | - | - | - | **-** | - | - | **-** | - | - | **-** |
| No majority | 35 | 32.6–37.4 | 37 | 34.6–39.4 | **<0.001** | 37 | 34.6–39.4 | **<0.001** | 37.6 | 35.2–40.0 | **<0.001** |
