# Supplementary material for: State and federal policies and school meal participation: A descriptive analysis from Arizona
Source: PLoS One. 2026 Jun 12;21(6):e0350416. doi: 10.1371/journal.pone.0350416 (PMC13262862; doi:10.1371/journal.pone.0350416)
Supplement: S3 Table — (DOCX) [file pone.0350416.s003.docx]

**S3 Table: Total Number of Breakfasts Served Daily (Adjusted)**

**A.** Total number of free breakfasts served daily across all time periods (Total, SE)

**B.** Total number of reduced-price breakfasts served daily across all time periods (Total, SE)

**C.** Total number of paid breakfasts served daily across all time periods (Total, SE)

The tables below show results derived from regression models analyzing total breakfasts served daily on average across multiple policy time periods (T0–T3), and eligibility categories.

***Model specification:*** Separate mixed-effects models with robust standard errors were run for each eligibility category (free, reduced-price, and paid). Each model controlled for school-level factors such as total enrollment and percentage of students eligible for free and reduced-price meals. Interaction terms between time period and locale, majority school race, and school level were included in the models to assess whether the effect of policy changes varied across these factors.

***Clustering:*** Standard errors were clustered at both the school and district level to account for within-group correlation.

***Dependent variables:*** ADP for breakfast (breakfasts served per day) for each eligibility category.

***Total meals served outcomes:*** Following model estimation, adjusted predictions were generated using the *predict* command, and totals were calculated using *total*. Statistical comparisons of means across time periods (T1, T2, T3 vs. T0) were conducted using *lincom*.

***Independent variables:*** Time period (T0, T1, T2, T3) and school-level factors, including school level, locale, majority school race, student enrollment, and proportion of students eligible for free and reduced-price meals. No transformations were applied to these variables.

***Observations:*** The number of observations differed based on eligibility category.

***Reported estimates:*** For each model, estimated means and 95% confidence intervals (CIs) or standard errors (SEs) are presented across policy periods, along with p-values testing differences from the baseline (T0).

*Regression outputs (coefficients, standard errors, R-squared, F-statistics) are available upon request.

**A.** Total number of free breakfasts served daily on average across all time period (Total, SE)- Adjusted

|  | **T0** | | **T1** | | | **T2** | | | **T3** | | |
| --- | --- | --- | --- | --- | --- | --- | --- | --- | --- | --- | --- |
|  | **(n=1,705)** | | **(n=1,700)** | | | **(n=1,700)** | | | **(n=1,699)** | | |
|  | **Total** | **SE** | **Total** | **SE** | **p-value (T1-T0)** | **Total** | **SE** | **p-value (T2-T0)** | **Total** | **SE** | **p-value (T3-T0)** |
| **Overall** | 814,853 | 11,058 | 856,033 | 11,124 | **0.009** | 939,333 | 11,203 | **<0.001** | 1,042,043 | 11,516 | **<0.001** |
| **Locale** |  |  |  |  |  |  |  |  |  |  |  |
| Rural | 106,088 | 3,590 | 106,757 | 3,561 | 0.895 | 125,540 | 3,662 | **<0.001** | 123,053 | 3,649 | **0.001** |
| Urban | 708,764 | 10,421 | 749,276 | 10,485 | **0.006** | 813,792 | 10,541 | **<0.001** | 918,990 | 10,824 | **<0.001** |
| **School Level** |  |  |  |  |  |  |  |  |  |  |  |
| Elementary | 643,140 | 9,131 | 675,321 | 9,159 | **0.013** | 720,774 | 9,285 | **<0.001** | 799,015 | 9,558 | **<0.001** |
| Middle | 65,192 | 2,743 | 67,498 | 2,776 | 0.555 | 71,594 | 2,788 | 0.102 | 81,111 | 2,904 | **<0.001** |
| High | 106,520 | 5,388 | 113,213 | 5,426 | 0.381 | 146,965 | 5,475 | **<0.001** | 161,917 | 5,567 | **<0.001** |
| **Majority school race** |  |  |  |  |  |  |  |  |  |  |  |
| White majority | 67,970 | 2,336 | 74,616 | 2,331 | **0.044** | 87,204 | 2,336 | **<0.001** | 97,908 | 2,334 | **<0.001** |
| Hispanic majority | 598,630 | 9,005 | 630,132 | 9,094 | **0.014** | 678,860 | 8,961 | **<0.001** | 763,089 | 9,046 | **<0.001** |
| AIAN majority | 47,885 | 1,841 | 44,292 | 1,859 | 0.17 | 49,660 | 1,883 | 0.5 | 45,953 | 1,859 | 0.46 |
| No majority | 100,368 | 2,889 | 106,994 | 2,867 | 0.104 | 123,609 | 2,883 | **<0.001** | 135,093 | 2,835 | **<0.001** |

**B.** Total number of reduced-price breakfasts served daily on average across all time period (Total, SE) - Adjusted

|  | **T0** | | **T1** | | | **T2** | | | **T3** | | |
| --- | --- | --- | --- | --- | --- | --- | --- | --- | --- | --- | --- |
|  | **(n=1,705)** | | **(n=1,700)** | | | **(n=1,700)** | | | **(n=1,699)** | | |
|  | **Total** | **SE** | **Total** | **SE** | **p-value (T1-T0)** | **Total** | **SE** | **p-value (T2-T0)** | **Total** | **SE** | **p-value (T3-T0)** |
| **Overall** | 80,341 | 1,187 | 90,477 | 1,204 | **<0.001** | 67,459 | 1140 | **<0.001** | 75,095 | 1,184 | **0.002** |
| **Locale** |  |  |  |  |  |  |  |  |  |  |  |
| Rural | 9,377 | 351 | 10,632 | 354 | **0.012** | 8,247 | 336 | **0.02** | 8,544 | 346 | 0.091 |
| Urban | 70,963 | 1,130 | 79,845 | 1,146 | **<0.001** | 59,212 | 1,087 | **<0.001** | 66,550 | 1,129 | **0.006** |
| **School Level** |  |  |  |  |  |  |  |  |  |  |  |
| Elementary | 61,720 | 975 | 68,017 | 994 | **<0.001** | 48,849 | 936 | **<0.001** | 54,665 | 979 | **<0.001** |
| Middle | 5,373 | 227 | 6,350 | 233 | **0.003** | 4,616 | 213 | **0.015** | 5,055 | 227 | 0.322 |
| High | 13,248 | 621 | 16,110 | 625 | **0.001** | 13,995 | 608 | 0.39 | 15,375 | 618 | **0.015** |
| **Majority school race** |  |  |  |  |  |  |  |  |  |  |  |
| White majority | 12,768 | 276 | 14,872 | 273 | **<0.001** | 11,083 | 277 | **<0.001** | 11,034 | 272 | **<0.001** |
| Hispanic majority | 54,535 | 983 | 60,566 | 991 | **<0.001** | 45,151 | 975 | **<0.001** | 53,057 | 980 | 0.287 |
| AIAN majority | - | - | - | - | - | - | - | - | - | - | - |
| No majority | 13,038 | 287 | 15,039 | 286 | **<0.001** | 11,222 | 293 | **<0.001** | 11,004 | 284 | **<0.001** |

**C.** Total number of paid breakfasts served daily on average across all time period (Total, SE) - Adjusted

|  | **T0** | | **T1** | | | **T2** | | | **T3** | | |
| --- | --- | --- | --- | --- | --- | --- | --- | --- | --- | --- | --- |
|  | **(n=1,705)** | | **(n=1,700)** | | | **(n=1,700)** | | | **(n=1,699)** | | |
|  | **Total** | **SE** | **Total** | **SE** | **p-value (T1-T0)** | **Total** | **SE** | **p-value (T2-T0)** | **Total** | **SE** | **p-value (T3-T0)** |
| **Overall** | 198,891 | 2,683 | 196,937 | 2,650 | 0.604 | 178,404 | 2,636 | **<0.001** | 176,786 | 2,612 | **<0.001** |
| **Locale** |  |  |  |  |  |  |  |  |  |  |  |
| Rural | 27,690 | 1,107 | 29,196 | 1,089 | 0.332 | 25,501 | 1,087 | 0.158 | 25,585 | 1,079 | 0.173 |
| Urban | 171,201 | 2,444 | 167,741 | 2,416 | 0.314 | 152,903 | 2,402 | **<0.001** | 151,201 | 2,379 | **<0.001** |
| **School Level** |  |  |  |  |  |  |  |  |  |  |  |
| Elementary | 156,472 | 2,287 | 152,302 | 2,246 | 0.193 | 136,705 | 2,253 | **<0.001** | 135,731 | 2,234 | **<0.001** |
| Middle | 13,162 | 500 | 13,169 | 487 | 0.992 | 11,753 | 486 | **0.043** | 11,974 | 475 | 0.085 |
| High | 29,257 | 1,235 | 31,466 | 1,261 | 0.211 | 29,946 | 1,239 | 0.693 | 29,081 | 1,229 | 0.92 |
| **Majority school race** |  |  |  |  |  |  |  |  |  |  |  |
| White majority | 47,882 | 1,167 | 52,321 | 1,183 | **0.008** | 47,764 | 1,168 | 0.943 | 47,435 | 1,149 | 0.785 |
| Hispanic majority | 111,509 | 2,096 | 104,162 | 2,105 | **0.013** | 94,150 | 2,088 | **<0.001** | 94,326 | 2,086 | **<0.001** |
| AIAN majority | - | - | - | - | - | - | - | - | - | - | - |
| No majority | 39,499 | 970 | 40,454 | 975 | 0.488 | 36,496 | 1,000 | **0.031** | 35,025 | 969 | **0.001** |
